# To Our Subscribers

**Published:** 1892-02

**Authors:** 


### Editorial.

### TO OUR SUBSCRIBERS.

In the January number allusion was made to the improvement of the circulation and of the material interests of this journal. We do not feel that this simple announcement should pass without further comment.

While the publishers of the International Dental Journal are in the constant endeavor with the instrumentalities of the Messrs. J. B. Lippincott Company, to produce a readable journal and to present the matter submitted to them in good form ; while all concerned are faithful and earnest in carrying out the intention of the promoters of an enterprise, entered into for the common good of the dental profession, it is natural for us to bring to our aid the readers of the journal. And we would add, as an opinion, these should not be satisfied until the whole of the readers of dental literature are counted among our subscribers. Therefore, it cannot be too much to ask each subscriber to interest himself in inducing others to place themselves on our lists. This request is not made in a begging spirit ; it is done because it is not unreasonable to desire all who are interested in dentistry to become associated with our effort. It is not too much to expect of the dental profession that it shall in the most liberal manner support its good journalism, and whether any person takes one or more dental periodicals, the obligation remains the same to join with the International Dental Journal. We do not attempt to induce such support because it is our sentimental interest to have it so, but for the reason that this journal is a faithful exponent of dentistry, and because we want arrayed with us every one who has a spark of professional loyalty actuating his life.

In the common callings, men stand shoulder to shoulder in support of movements for the common good of their guild, and certainly in dentistry we have reasonable hope for even more interest and more substantial and enduring encouragement.

Therefore, with a feeling of the fitness of things, we want all to be our coadjutors.

